# YY super sperm lead to all male triploids and tetraploids

**DOI:** 10.1186/s12863-015-0230-z

**Published:** 2015-06-25

**Authors:** Rong Zhou, Jun Xiao, Qinbo Qin, Bin Zhu, Rurong Zhao, Chun Zhang, Min Tao, Kaikun Luo, Jing Wang, Liangyue Peng, Shaojun Liu

**Affiliations:** Key Laboratory of Protein Chemistry and Developmental Biology of the State Education Ministry of China, College of Life Sciences, Hunan Normal University, Changsha, Hunan People’s Republic of China, 410081

**Keywords:** Androgenesis, Super male crucian carp, Unreduced gametes, All male triploids, All male tetraploids, Carp

## Abstract

**Background:**

Androgenesis is a unique and rarely encountered reproductive mode in which the offspring only inherit the paternal nuclear genome, resulting in relatively few viable individuals.

**Results:**

In this study, a super male (YY) crucian carp was obtained by androgenesis with the diploid sperm of autotetraploid crucian carp (4*n* = 200). Flow cytometry assay confirmed the fish was diploid. The scanning electron microscopy and flow cytometry analysis results of sperm revealed that the YY crucian carp produced unreduced diploid sperm. To prove the special reproductive characteristic and homozygosity of the YY crucian carp, three rounds of hybridization experiments were performed. First, self-crossing between female androgenic progenies and YY crucian carp generated all male tetraploids. Then, hybridization of female red crucian carp (2*n* = 100) and female autotetraploid fish (4*n* = 200) with YY crucian carp produced all male triploids and all male tetraploids, respectively.

**Conclusions:**

This is the first time reported producing a viable diploid homozygous YY fish with unreduced diploid sperm of the autotetraploid fish, which were derived from distant hybridization. These results will not only help explaining the sex determination mechanism in teleost fish, but also play a significant role in genetic breeding in aquaculture.

## Background

Androgenesis is a uniparental developmental mode, in which the offspring only inherit the paternal nuclear genome making them clones of the father [1]. This mode of reproduction has been successfully carried out in several fish species that adopt external fertilization, such as *Cyprinus carpio* [2], *Oncorhynchus mykiss* [3, 4], *Oreochromis niloticus* [5], and *Misgurnus anguillicaudatus* [6]. Androgenesis is one of the best chromosome manipulation techniques available and is useful in constructing pure genetic lines, generating monosexual fish [5, 7], restoring endangered or extinct species from cryopreserved spermatozoa [8], and evaluating how the mitochondrial genome affects development [9].

In general, embryonic development in teleost fish is activated by fertilization. In androgenesis, an egg whose nuclear content is destroyed serves as the vector; it is then fertilized with an untreated native sperm to form a haploid embryo. The egg provides the functional cytoplasm, but does not contribute to the nuclear genome of the offspring during development. Subsequently, to obtain diploid embryos, the first cell division is inhibited by either heat or pressure shock. After the first cell division blockage, the genome is duplicated to form a diploid. There are two physical treatments in the entire process. This first is inactivating eggs by ultraviolet irradiation or gamma ray, the second is preventing first mitosis by heat or pressure shock. Researchers have compared the effects of both treatments on embryo survival rates. Thorgaard *et al.* found that the survival rate of androgenic progenies with diploid sperm is much higher than those with haploid sperm, which indicates that pressure shock treatment and/or homozygosity may be responsible for the low survival rate of androgenic double haploids; while the ultraviolet irradiation inactivation method did not pose much of a problem [10]. Yamaha *et al.* found that both heat and pressure shock induced developmental disorders during embryonic development, in particular they suppressed dorso-ventral differentiation [11]. To date, few examples of successful androgenesis with diploid sperm have been reported for limited kinds of fertile tetraploid fishes [1, 6].

Researchers have reported the first allotetraploid hybrids (4*n* = 200, abbreviated as 4*n*AT) derived from crossing between red crucian carp (*Carassius auratus* red var.) (2*n* = 100, abbreviated as RCC) (♀) and common carp (*Cyprinus carpio* L.) (2*n* = 100, abbreviated as CC) (♂). Since then, a consecutive allotetraploid line has been established from generations F_3_ to F_24_ [12, 13]. Recently, autotetraploid crucian carp (4*n* = 200) have been obtained by distant hybridization between RCC (2*n* = 100) (♀) and blunt snout bream (*Megalobrama amblycephala*) (2*n* = 48, abbreviated as BSB) (♂); the autotetraploid crucian carp line was formed from generations F_2_ to F_8_ [14]. Both tetraploid lines are useful for genetic research and breeding, especially androgenesis, because tetraploids can produce many diploid spermatozoa. Sun *et al.* induced androgenesis in diploid 4*n*AT sperm and successfully yielded bisexual fertile diploid AT-ag [15]. Unfortunately, all of the male AT-ag fish obtained by Sun *et al.* were heterozygous for both the sex ratios of male to female fish in F_1_ ag × ag and AT × AT-ag did not differ significantly from the expected ratio of 1:1 [15].

In this study, we used the diploid spermatozoa of autotetraploid crucian carp to artificially induce androgenesis to screen homozygous super male fish. In the androgenetic offspring, a male fish with white sperm was selected. The following characteristic analysis revealed that the YY crucian carp was diploid and produced unreduced diploid sperm. Furthermore, self-crossing and hybridization of the YY crucian carp with diploid female RCC and 4*n*AT sired all male triploids and tetraploids, respectively, which proves that the male fish selected was a homozygous super-male YY crucian carp. This is the first report in which a viable homozygous super-male fish with diploid sperm was obtained by distant hybridization and androgenesis. This fish will benefit the study of the sex determination mechanism in teleost fish, and producing all male fish making it important for genetic breeding in aquaculture.

## Results

### The formation of diploid androgenetic fish

**Fig. 1:**
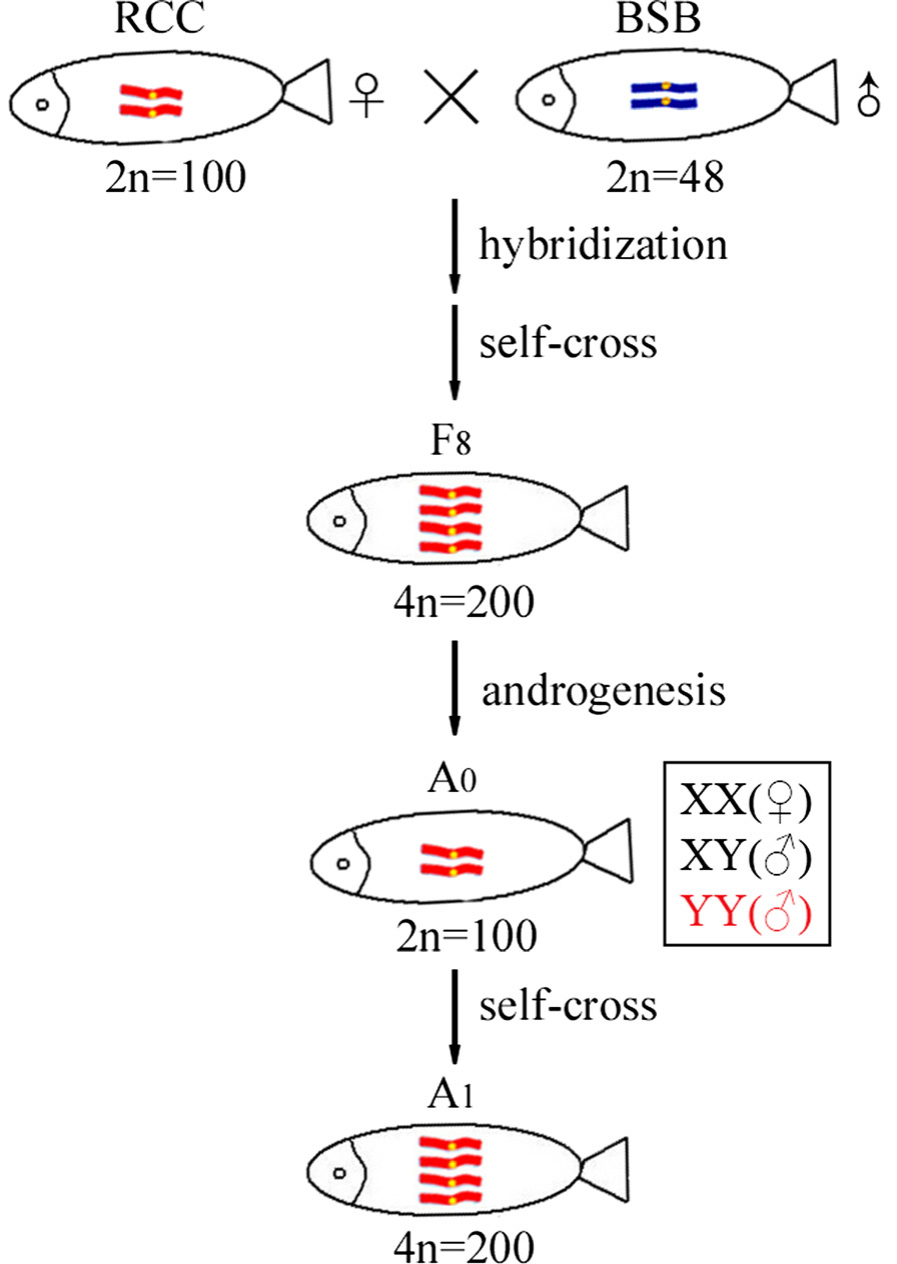
The origin and formation of androgenetic fish. The RCC and BSB chromosomes are red and blue, respectively. The probable sex and sex chromosome compositions of the A_0_ individuals are listed in the box

A both female and male fertile autotetraploid crucian carp line, obtained via the intergeneric hybridization of RCC (2*n* = 100, ♀) × BSB (2*n* = 48, ♂) and self-crossing of F_1_, has been established from F_2_ to F_8_ [14]. Diploid spermatozoa with a large diameter of approximately 2.4 μm were generated by the male autotetraploid crucian carp [14]. In the present study, artificial androgenesis was performed using ultraviolet irradiation inactivated RCC eggs as the recipient and untreated diploid spermatozoa from the autotetraploid crucian carp as the donor. Diploid embryos were formed directly after fertilization and subsequently developed into androgenetic fish. During androgenesis in this study, the fertilization rate is about 10.01 %, the hatching rate about 2.10 %, and the survival rate about 1.66 % (Table 1). The survival rate has improved obviously by using the diploid sperm when compared to previous studies using haploid sperm in *O. mykiss* (0.8 %) [10]. This androgenetic fish population was named as A_0_ (Fig. 1), both the male and female fish were fertile. A self-cross between male and female A_0_ individuals obtained A_1_ individuals (Fig. 1).

**Table 1:** Statistic analysis of androgenesis with diploid sperm

Eggs	2047
Fertilization embryos	205
Fertilization rate	10.01 %
Hatching fry	43
Hatching rate	2.10 %
Adult fish	34
Survival rate	1.66 %

Fertilization rate = (number of fertilization embryos/number of eggs) × 100 %

Hatching rate = (number of hatching fry/number of eggs) × 100 %

Survival rate = (number of adult fish / number of eggs) × 100 %

### Morphological and genetic characteristics of super-male (YY) crucian carp

**Fig. 2:**
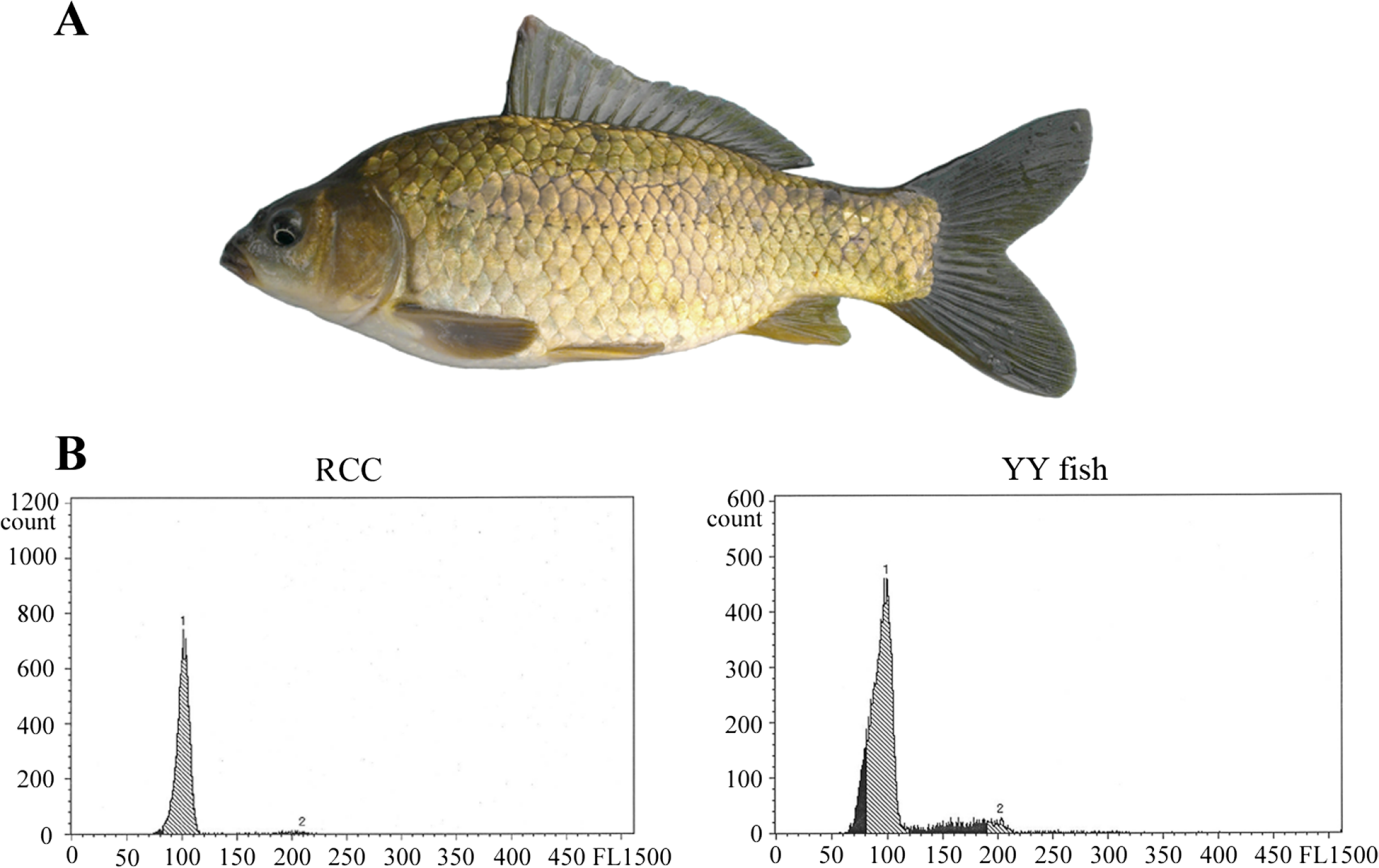
Morphology and ploidy analysis of the YY fish. **a** The appearance of the YY fish. **b** DNA content means of RCC and YY fish were measured by flow cytometry analysis. RCC was used as the control

Theoretically, the androgenetic progenies contained three genotypes (XX, XY and YY). Actually, all the androgenetic progenies displayed the similar phenotype. We cannot distinguish the three fishes via comparing phenotypes. In the A_0_ population, a special male fish with white semen was distinguished from other male fish with water-like semen. We therefore presumed that this was a homozygous super-male fish and named it YY crucian carp. The body of the YY crucian carp was gray in color, which was consistent with its male parent the male autotetraploid crucian carp [14] (Fig. 2a). Regarding the measurable traits, the YY crucian carp exhibited higher body length to width, head length to width, and tail length to width ratios; whereas it had a lower body width to head width ratio than RCC and the F_8_ autotetraploid crucian carp (Table 2). As for the countable traits, the YY crucian carp possessed more lateral and upper lateral scales, and less abdominal fins than the RCC and F_8_ autotetraploid crucian carp, while there was no obvious difference in the other traits (Table 3). To study the genetic characteristics of the YY crucian carp, flow cytometry analysis was used to measure the DNA content of the YY crucian carp. The YY crucian carp had a DNA content mean of 100, which was identical to that of the RCC control (Fig. 2b). Therefore, both the larger sperm size of the autotetraploid crucian carp and ploidy observations via chromosome spread and flow cytometry analysis indicated that the YY crucian carp was diploid.

**Table 2:** Comparison of RCC, F_8_, and YY fish measurable traits

Fish type	Whole length/body length	Body length/body width	Body length/head length	Head length/head width	Tail length/tail width	Body width/head width
RCC	1.22 ± 0.02	2.18 ± 0.02	3.72 ± 0.03	1.07 ± 0.03	0.82 ± 0.03	1.84 ± 0.03
F_8_	1.23 ± 0.02	2.23 ± 0.08	3.73 ± 0.02	1.08 ± 0.02	0.84 ± 0.02	1.88 ± 0.06
YY fish	1.21 ± 0.02	2.82 ± 0.04	3.87 ± 0.02	1.25 ± 0.02	0.87 ± 0.02	1.72 ± 0.03

**Table 3:** Comparison of RCC, F_8_, and YY fish countable traits

Fish type	No. of lateral scales	No. of upper lateral scales	No. of lower lateral scales	No. of dorsal fins	No. of abdominal fins	No. of anal fins
RCC	29.20 ± 0.70 (28–30)	5.60 ± 0.50 (5–6)	5.70 ± 0.47 (5–6)	III + 18.65 ± 0.49 (III + 18-19)	8.55 ± 0.51 (8–9)	III + 5.65 ± 0.49 (III + 5-6)
F_8_	29.54 ± 1.03 (29–32)	5.36 ± 0.50 (5–6)	6.81 ± 0.75 (5–7)	III + 18.27 ± 0.46 (III + 18-19)	8.63 ± 0.50 (8–9)	III + 5.45 ± 0.52 (III + 5-6)
YY fish	33.54 ± 0.90 (33–34)	5.72 ± 0.35 (5–6)	5.81 ± 0.82 (5–7)	III + 18.43 ± 0.51 (III + 18-19)	8.21 ± 0.42 (8–9)	III + 5.34 ± 0.46 (III + 5-6)

### The reproductive features of the super-male (YY) crucian carp

**Fig. 3:**
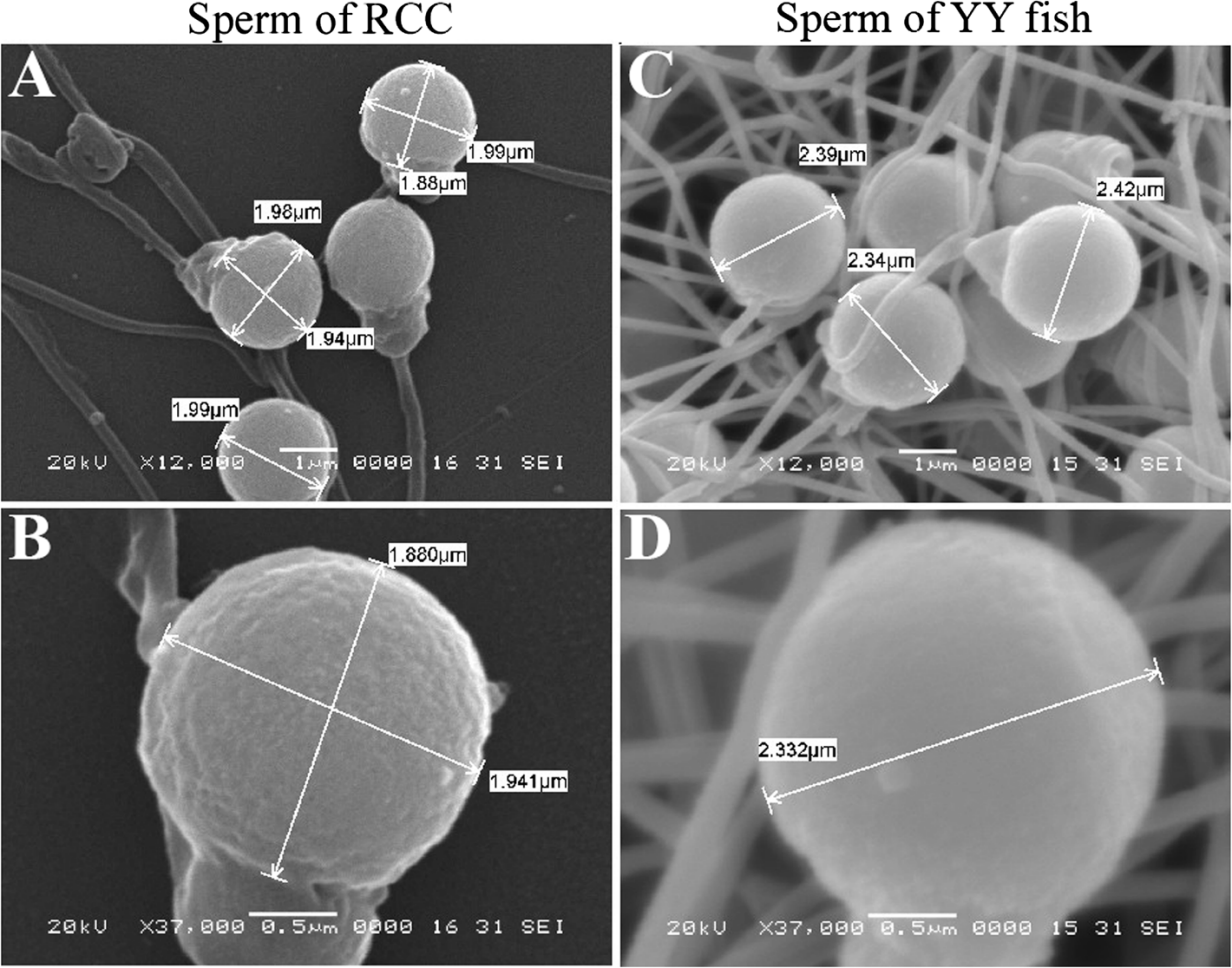
YY fish spermatozoa morphology. **a** and **b** RCC spermatozoa at different magnification scales. **c** and **d** YY fish spermatozoa at different magnification scales. The magnification and scale bar are labeled in the pictures. Sperm diameter is also indicated

**Fig. 4:**
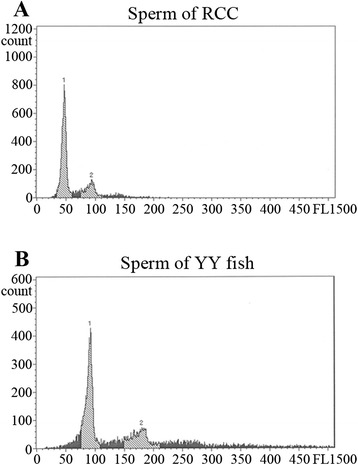
DNA content analysis of YY fish spermatozoa. DNA content analysis of sperm from the RCC (a)and YY fish (b) was performed by flow cytometry. Sperm of RCC was used as the control

The 2-year-old YY crucian carp produced white semen, while the 1-year-old fish did not. This indicated that the YY crucian carp reaches sexual maturity at 2 years of age. The microstructure of the mature YY crucian carp sperm was observed via the scanning electron microscope. RCC spermatozoa were haploid with a diameter range of 1.88–1.99 μm as standard (Fig. 3a and b). In contrast, the YY crucian carp spermatozoa ranged from 2.33 to 2.42 μm in diameter (Fig. 3c and d), which is larger than the haploid control spermatozoa but similar to the autotetraploid crucian carp diploid spermatozoa (~2.40 μm diameter) [14]. The DNA content measurement of sperms by flow cytometry analysis revealed that the DNA content of sperm from the YY crucian carp is the double of sperm from RCC (Fig. 4a and b). The above results both proved that the diploid YY crucian carp produced unreduced diploid sperm.

### Self-cross between female A_0_ individuals and the YY crucian carp sired all-male tetraploid progenies

**Fig. 5:**
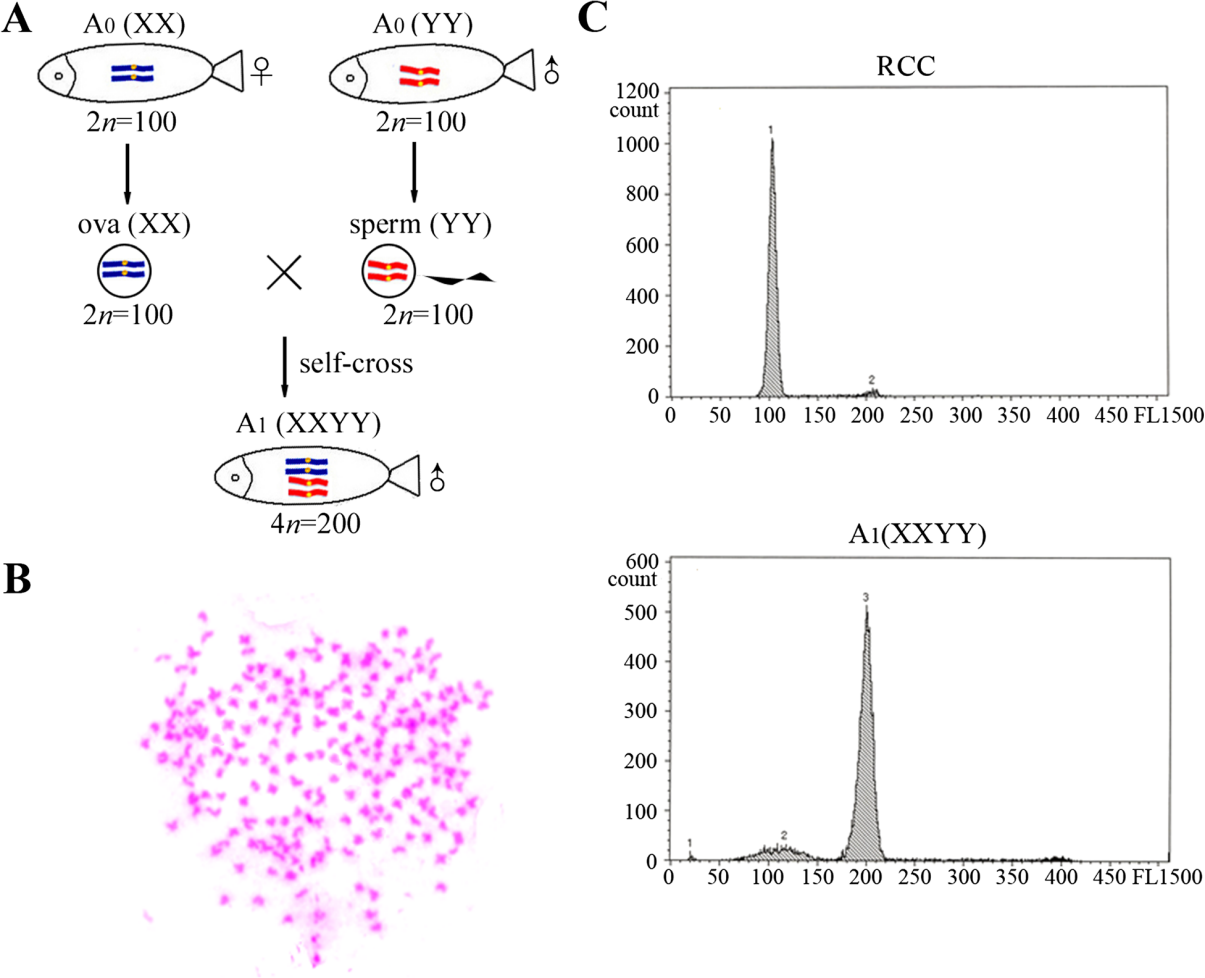
Self-cross between female androgenetic fish and the YY fish. **a** Self-cross between female diploid androgenetic fish (A_0_) and male YY fish. **b** Chromosome observations of A_1_ individual. **c** Ploidy analysis of A_1_ fish by flow cytometry. RCC was used as the standard. The X and Y chromosomes are blue and red, respectively

To estimate the fertility, homozygosity, and gamete ploidy of the putative YY crucian carp, a self-cross between the female diploid androgenetic fish (A_0_) and the YY crucian carp was performed. The cross and presumed sexual chromosome composition are shown in Fig. 5a. All of the offspring were male. Moreover, both the male and female A_0_ individuals produced diploid gametes, thus, siring tetraploid offspring (Fig. 5a). Chromosome observations of metaphase spreads confirmed that the chromosome number of self-cross offspring (A_1_) was 200 (Fig. 5b). DNA content measurement by flow cytometry analysis revealed that the DNA content of A_1_ fish was double that of RCC (Fig. 5c).

### Hybridization between different ploidy female fish and super-male (YY) crucian carp

**Fig. 6:**
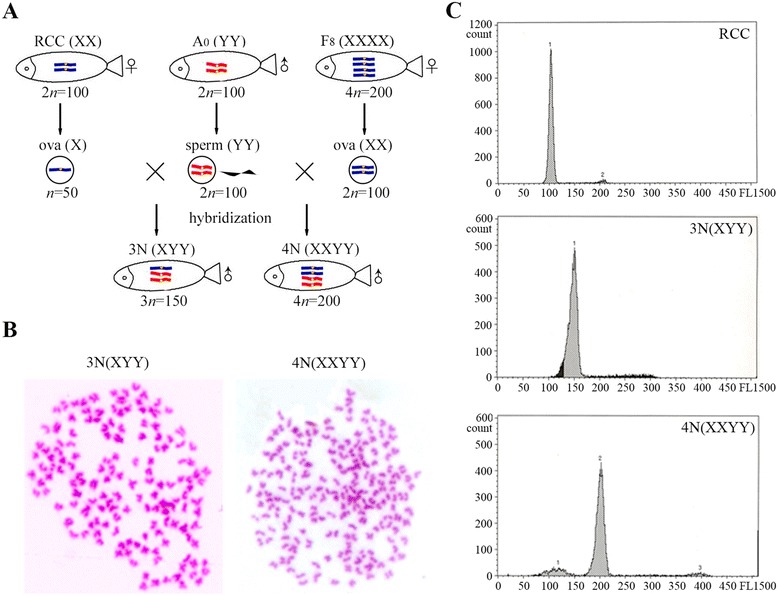
Hybridization between female RCC (diploid), 4*n*AT (tetraploid), and YY fish. **a** The summarized hybridization schedule. **b** Chromosome observations of the all-male triploids and tetraploids. **c** DNA content measurement of the all-male triploids and tetraploids. RCC was used as the control

To further confirm the ploidy of YY super sperm and the homozygosity of the YY crucian carp, hybridizations between different ploidy female fish and YY crucian carp were performed. The hybridization plans were shown in Fig. 6a. The composition of proposed sexual chromosomes in each fish was described (Fig. 6a). Both hybridization groups sired all-male offspring. As stated before, RCC produced haploid ova, while the autotetraploid crucian carp generated diploid ova. Therefore, hybridizations between RCC or autotetraploid crucian carp and the YY crucian carp obtained triploid and tetraploid progenies, respectively. Chromosome observations revealed that the chromosome number of the triploid offspring was 150, and that of the tetraploids was 200 (Fig. 6b). Flow cytometry analysis revealed that the DNA content of triploid offspring was one half that of RCC and that of tetraploids was two-fold (Fig. 6c). All of the results prove that the YY crucian carp was homozygous and produces unreduced diploid sperm.

## Discussion

Androgenesis is an induced developmental process whereby only the paternal nuclear genome is inherited, it involves an inactivated egg and an active sperm for fertilization [16]. To evaluate the inactivation of eggs and verify the androgenetic nature of the offspring, several methods have been developed to distinguish double haploid individuals and hybrid fish of the same ploidy. For example, random amplification polymorphic DNA (RAPD) analysis has been used in *Hemigrammus caudovittatus* [17], recessive color markers in *Oreochromis niloticus* [18], and allozyme genotypes in *Misgurnus anguillicaudatus* [6]. In this study, androgenesis involved the sperm of autotetraploid crucian carp and the ova of RCC. As previously described, the autotetraploid crucian carp produced diploid gametes, while the RCC produced haploid ova. The interploid crossing generated triploids [14]. Therefore, the chromosome number and DNA content irrefutably distinguish A_0_ individuals from female RCC and F_8_ male autotetraploid hybrids. The chromosome counting and ploidy analysis results revealed that the YY crucian carp was diploid not triploid, which verified the androgenetic nature of the YY crucian carp.

Qin *et al*. have reported that autotetraploid crucian carp were obtained by distant hybridization, and contained four sets of RCC chromosomes [14]. Thus, the diploid sperm produced by the autotetraploid crucian carp should contain two sets of RCC chromosomes. However, they also pointed out that some measurable and countable phenotypic changes occurred in autotetraploid crucian carp compared with RCC, e.g., the gray body color and barbells [14]. Although the autotetraploid crucian carp differs from the interspecific allotetraploid hybrid fish, genome variation still occurred during whole genome duplication. Based on the genetic background of the autotetraploid crucian carp, the diploid sperm proved to be a hybrid gamete. Moreover, the gray body color of YY crucian carp is consistent with the autotetraploid crucian carp and differs from RCC.

Generally speaking, the fertile diploid fish produces haploid gametes through normal meiosis. However, the diploid hybrid fish, which contains two different sets of chromosomes, would meet disordered synapsis of homologous chromosomes via normal meiosis. This difficulties could be rescued by mechanisms as follows: (i) pre-meiotic doubling of DNA during spermatogenesis [19]; (ii) fusion of sperm after meiosis [20]; and (iii) abortive cytokinesis during meiosis [21]. Therefore, the diploid hybrid fish derived from distant hybridization usually produces unreduced diploid gametes. This phenomenon has been observed in studies of Cherfas *et al.* and Liu *et al.* [22–24]. Interestingly, in this study, DNA content analysis on adult fish and sperm size and DNA content measurements also revealed that the androgenetic YY crucian carp was diploid and produced unreduced diploid gametes. While the extract genetic mechanism related to the unreduced diploid gametes is still unknown. For the survival advantages of androgenesis using diploid gametes, the unreduced gametes from the hybrid fish would be benefit to establish a pure clonal line. For example, the gynogenetic offspring of 4*n*AT can produce unreduced diploid ova, and forms a diploid gynogenetic clonal line from G_0_ to G_10_ till now [24].

In mammals, sex determination is generally due to male heterogamety (XY system). The master sex determining gene SRY, which is located on the Y chromosome, is identified [25, 26]. In fish species, for its special position in evolution, the sex determination system is complex and diverse [27]. Both male (males are XY or YY and females are XX) and female heterogamety (females are ZW and males are ZZ) occur. More complicated systems can involve multiple sex chromosomes and multiple gene loci on autosomes, and also environmental factors (for example temperature) [16]. Until now, morphologically distinct sex chromosomes and key sex determining genes have not been identified. Unisexual reproduction pathways, such as gynogenesis and androgenesis, are excellent genetic tools to explore the sex determination mechanism [28]. In common carp, sex determination is thought to be an XX/XY system [2]. In RCC and common carp hybrids, gynogenesis with diploid 4*n*AT ova sired all female progenies [24]. While androgenesis with diploid 4*n*AT sperm sired both male and female offspring [15], proving the presence of an XX/XY sex determination system. Here, androgenesis with diploid sperm from autotetraploid crucian carp produced male and female individuals, providing additional evidence on the sex determination system in crucian carp.

In allopolyploids, abnormal paring and segregation of chromosomes during meiosis, which leads to the production of gametes with complete paternal or maternal chromosomes, has been observed [29]. The androgenetic male fish produce unreduced diploid gametes with two rounds of genome duplication. Thus, in the gamete generation process, XY male fish can produce sperm with three different genotypes (XX, XY, and YY). After crossing with female fish, the offspring should segregate as XXXX female and XXXY/XXYY male progenies. In contrast, YY male fish only produce YY genotypical sperm. All male offspring were expected from the crosses. In practice, the putative YY crucian carp mating with either diploid or tetraploid female fish yielded all male triploids and tetraploids, respectively. This result is consistent with the latter assumption. Thus, the homozygous YY nature of the putative super male fish was confirmed by crossing them with female fish of different ploidy and genetic background. We observed that the super male YY crucian carp produced white semen, while other male fish just produced water-like semen. Kirankumar *et al.* found that the YY male fish displayed higher sexual gland index (GSI) and sperm counts than XY tiger barb male fish [30]. The male androgenetic fish with water-like semen have also been used for hybridization with diploid female red crucian carp to evaluate the genotype by progeny testing. The offspring sex of male A_0_ with water-like semen includes male and female (data not shown). This result is consistent with the observation of previous studies [30].

## Conclusions

This is the first report in which homozygous super male YY crucian carp are obtained via distant hybridization and androgenesis. Regarding the unreduced sperm, the YY crucian carp could maintain the fish clonal line via androgenesis without any treatments for chromosome duplication. In theory, the homozygous YY crucian carp provides a perfect material for exploring the major sex determination-related genes to artificially switch sex differentiation. It also provides a platform to explore the mechanism behind unreduced sperm production. In aquaculture breeding, the YY crucian carp is available for the production of all male tetraploids, which not only benefits the study of the ploidy mechanism in fish, but also provides male parents for producing large numbers of all male sterile triploids.

## Methods

### Ethics

All samples were cultured in ponds and fed with artificial feed at the Protection Station of Polyploidy Fish, Hunan Normal University. Fish treatments were carried out according to the Care and Use of Agricultural Animals in Agricultural Research and Teaching, approved by the Science and Technology Bureau of China. For the fish in question are not rare or near extinction (first-class or second-class state protection level), approval from the Department of Wildlife Administration is not required for the experiments conducted in this paper.

### Experimental fish and crossing

Androgenesis was performed according to previously described methods, with some modification [15, 31]. During the reproductive season (from April to June), one mature male autotetraploid F_8_ fish were selected to produce diploid sperm. Semen was collected and diluted with Hank’s buffer (1:4, v/v). About 2047 eggs from one RCC were spread in a monolayer on three independent glass petri dishes containing synthetic ovarian fluid, and then irradiated by 300 mJ/cm^2^ ultraviolet rays for 180 s with constant stirring to ensure a homogenous inactivation of all eggs. After irradiation, the eggs were inseminated with untreated milt and hatched at 25 °C to form the androgenetic individuals. The fertilization rate, hatching rate and survival rate have been calculated.

For the self-crosses and interploid hybridization, the mature eggs of genetically different female fish, including an A_0_ individual, RCC, and autotetraploid crucian carp F_8_ were fertilized with mature sperm from the YY crucian carp *in vitro* during the reproductive season. The embryos were hatched under the conditions previously mentioned. For progeny testing, during the reproductive season, ten one-year-old offspring were selected randomly and dissected to determine sex. The rest five hundred offspring in each hybridization group were squeezed semen artificially to identify sex.

### Morphological measurements

The morphological and countable characteristics of the YY crucian carp were examined at two years of age as previously described [32]. The same characteristics in RCC and F_8_ in Tables 1 and 2 are cited for comparison [14].

### Flow cytometry analysis

DNA contents of the YY crucian carp, A_1_ offspring, all male triploids, and all male tetraploids were measured via flow cytometry analysis to determine the ploidy of each fish. First, approximately 0.5 mL of blood was collected from the caudal vein of each fish using a syringe containing 100 units of sodium heparin. The blood samples were then diluted with ACD buffer to a proper concentration and stained with DAPI (Sigma) at room temperature for 10 min. Following filtration, DNA content measurement was performed in a Partec Cell Counter Analyzer.

DNA contents of the sperm of the YY crucian carp and RCC were analyzed by similar ways with blood cells. Sperms were collected from each fish and diluted with PBS buffer to an appropriate concentration, followed by stained with DAPI, filtration, and measured by the Partec Cell Counter Analyzer.

### Chromosome counting

The chromosomes of 12-month-old fish were prepared by peripheral blood cell cultivation according to the procedure reported by Xiao *et al.* [33]. For each fish, approximately 0.3 mL blood was collected with a syringe containing 0.2 mL 0.1 % sodium heparin. The blood cells were then cultured in RPMI-1640 (Invitrogen) supplemented with 15 % calf serum (Invitrogen) and incubated in a 5 % CO_2_ environment at 26° for 68–72 h. Colchicine was added to the medium for a 4 h incubation. The cells were harvested and hypotonically treated with 0.075 M KCl and then fixed in methanol-acetic acid (3:1, v/v) overnight with three changes. Chromosomes were prepared via dropping and air drying, then stained in 4 % Giemsa solution for 30 min, and finally examined microscopically.

### Spermatozoa phenotype

The phenotype of spermatozoa was scanned according to Qin *et al*.[14]. Briefly, RCC and YY crucian carp semen was collected and fixed with 2.5 % glutaraldehyde solution, then with 1 % osmic acid solution. After dehydration, dropping, and desiccation, the sperm samples were subjected to atomized gilding for scanning electron microscopy (X-650 scan-electron micro-scope, Hitachi, Germany).

### Statistical analysis

Analysis of variance and pairwise comparisons of the data were analyzed in SPSS Statistics 17.0.

## Abbreviations

RCC: Red crucian carp
4nAT: The allotetraploid hybrids
CC: Common carp
BSB: Blunt snout bream
